# Supplementary material for: Long-Lasting Anti-Inflammatory and Antinociceptive Effects of Acute Ammonium Glycyrrhizinate Administration: Pharmacological, Biochemical, and Docking Studies
Source: Molecules. 2019 Jul 4;24(13):2453. doi: 10.3390/molecules24132453 (PMC6651237; doi:10.3390/molecules24132453)
Supplement: Supplementary file 1 [file molecules-24-02453-s001.pdf]

Supplementary Material

Long-Lasting Anti-Inflammatory and Antinociceptive Effects of Acute Ammonium Glycyrrhizinate Administration: Pharmacological, Biochemical, and Docking Studies

Francesco Maione [1,†], Paola Minosi [2,†], Amalia Di Giannuario [2], Federica Raucci [1], Maria Giovanna Chini [3], Simona De Vita [3], Giuseppe Bifulco [3], Nicola Mascolo [1] and Stefano Pieretti [2,*]

1 Department of Pharmacy, School of Medicine and Surgery, University of Naples Federico II, Via Domenico Montesano 49, 80131 Naples, Italy
2 National Centre for Drug Research and Evaluation, Istituto Superiore di Sanità, Viale Regina Elena 299, 00161 Rome, Italy
3 Department of Pharmacy, University of Salerno, Via Giovanni Paolo II 132, 84084 Fisciano (SA), Italy
* Correspondence: stefano.pieretti@iss.it; Tel.: +39-0649902451
† These authors contributed equally to this work.

FLAP

We performed molecular docking experiment on the 5-lipoxygenase–activating protein (FLAP), known for activating the conversion of arachidonic acid into 5-(*S*)-hydroperoxy-6,8,11,14-eicosatetraenoic acid (5-HpETE) and leukotriene A_4 (LTA_4) [1]. The crystal structure of the human FLAP bound to a known inhibitor (PDB: 2Q7M) [1] was used during computational studies by software Glide [2–4]. The grid center was set on the ligand (X = 65.86, Y = 83.68, and Z = 31.92) and the inner box was extended for 24 Å in the three dimensions. The molecular docking (Figure S1) of both MK-591 and AG was carried out in the XP mode saving 10,000 poses and the best 800 poses were minimized. Afterwards, a postdocking optimization was carried out, setting the rejection cut-off at 0.8 kcal/mol, and a maximum of 30 poses per ligand.

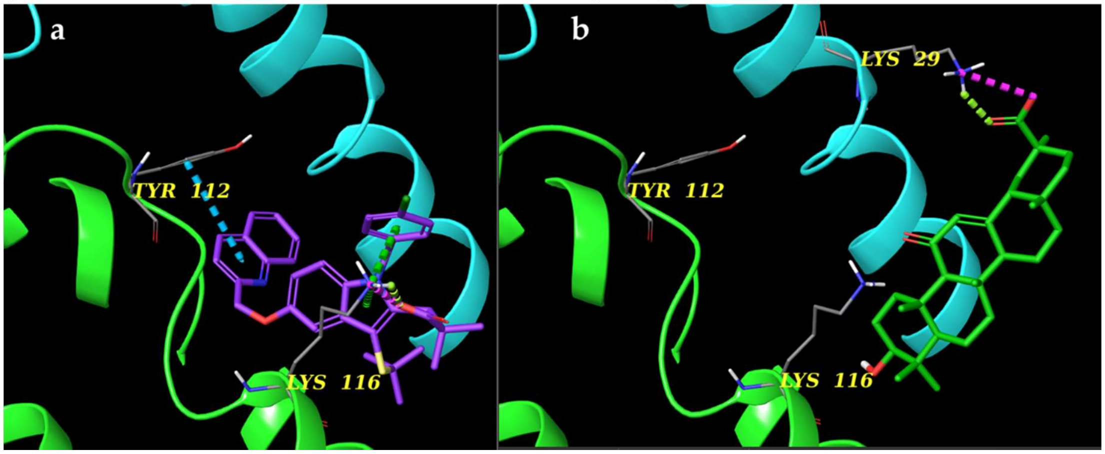

Figure 1. (**a**) 3D models of the inhibitor MK-591 (colored by atom types: C purple, O red, S yellow, N blue) and (**b**) AG (colored by atom types: C green, O red, polar H white) in the binding site of 5-lipoxygenase–activating protein (FLAP). Protein chains are A and B are reported in green and cyan (chain C not shown). Light green dotted lines represent H-bonds, dark green dotted lines are π-cation interactions, pink dotted lines represent salt bridges, and cyan dotted lines are π-π stacking. Important residues are labelled in yellow.

Comparing the binding mode of AG with respect to the co-crystallized inhibitor, MK-591 forms several interactions with Lys116 and a π-π interaction with Tyr112 (Figure S1a) essential for the inhibitory activity. On the other hand, AG was not able to interact with these key amino acids; the only bonds formed are with Lys29, far from the binding cavity (Figure S1b). The results suggested a non-optimal binding between AG and FLAP.

References

1. Ferguson, A.D.; McKeever, B.M.; Xu, S.; Wisniewski, D.; Miller, D.K.; Yamin, T.-T.; Spencer, R.H.; Chu, L.; Ujjainwalla, F.; Cunningham, B.R.; et al. Crystal Structure of Inhibitor-Bound Human 5-Lipoxygenase-Activating Protein. *Science*. **2007**, *317*, 510–512, doi:10.1126/science.1144346
2. Friesner, R.A.; Murphy, R.B.; Repasky, M.P.; Frye, L.L.; Greenwood, J.R.; Halgren, T.A.; Sanschagrin, P.C.; Mainz, D.T. Extra Precision Glide: Docking and Scoring Incorporating a Model of Hydrophobic Enclosure for Protein-Ligand Complexes. *J. Med. Chem*. **2006**, *49*, 6177–6196, doi:10.1021/jm051256o.
3. Halgren, T.A.; Murphy, R.B.; Friesner, R.A.; Beard, H.S.; Frye, L.L., Pollard, W.T.; Banks, J.L. Glide: A New Approach for Rapid, Accurate Docking and Scoring. 2. Enrichment Factors in Database Screening. *J. Med. Chem*. **2004**, *47*, 1750–1759, doi:10.1021/jm030644s.
4. Friesner, R.A.; Banks, J.L.; Murphy, R.B.; Halgren, T.A.; Klicic, J.J.; Mainz, D.T.; Repasky, M.P.; Knoll, E.H.; Shaw, D.E.; Shelley, M.; et al. Glide: A New Approach for Rapid, Accurate Docking and Scoring. 1. Method and Assessment of Docking Accuracy. *J. Med. Chem*. **2004**, *47*, 1739–1749, doi:10.1021/jm0306430.
